# New-onset cardiovascular diseases post SARS-CoV-2 infection in an urban population in the Bronx

**DOI:** 10.1038/s41598-024-82983-7

**Published:** 2024-12-28

**Authors:** Jason Y. Lu, Justin Y. Lu, Stephen H. Wang, Katie S. Duong, Wei Hou, Tim Q. Duong

**Affiliations:** 1 Department of Radiology, Albert Einstein College of Medicine and Montefiore Medical Center, Bronx, New York, USA; 2 Department of Surgery, Beth Israel Deaconess Medical Center, Harvard Medical School, Boston, MA USA; 3 Center for Health & Data Innovation, Albert Einstein College of Medicine and Montefiore Medical Center, Bronx, New York, USA; 4 Albert Einstein College of Medicine and Montefiore Medical Center, 1300 Morris Park Avenue, Bronx, New York, 10461 USA

**Keywords:** myocarditis, stroke, heart attack, long COVID-19, post-acute sequelae of COVID-19 (PASC), Epidemiology, Cardiology

## Abstract

This study investigated the incidence of new-onset cardiovascular disorders up to 3.5 years post SARS-CoV-2 infection for 56,400 individuals with COVID-19 and 1,093,904 contemporary controls without COVID-19 in the Montefiore Health System (03/11/2020 to 07/01/2023). Outcomes were new incidence of major adverse cardiovascular event (MACE), arrhythmias, inflammatory heart disease, thrombosis, cerebrovascular disorders, ischemic heart disease and other cardiac disorders between 30 days and (up to) 3.5 years post index date. Results were also compared with a pre-pandemic cohort over similar observation duration (*N* = 64,541). Cumulative incidence and hazard ratios adjusted for competitive risks were analyzed. Compared to contemporary controls, hospitalized COVID-19 patients had significantly higher risk of developing MACE (aHR = 2.29, 95% confidence interval [2.27, 2.31], *p* < 0.001), arrhythmias (aHR = 2.54[2.50, 2.58], *p* < 0.001), inflammatory heart disease (aHR = 5.34[4.79, 5.96], *p* < 0.001), cerebrovascular (aHR = 2.05[2.00, 2.11], *p* < 0.001), other cardiac disorders (aHR = 2.31[2.26, 2.35], *p* < 0.001), thrombosis (aHR = 4.25[4.15, 4.36], *p* < 0.001), and ischemic heart disease (aHR = 1.89[1.86, 1.92], *p* < 0.001). Non-hospitalized COVID-19 patients had slightly higher risk of developing MACE (aHR = 1.04[1.03, 1.06], *p* < 0.001), arrhythmias (aHR = 1.10[1.08, 1.12], *p* < 0.001), inflammatory heart disease (aHR = 2.29 [2.03, 2.59], *p* < 0.001), cerebrovascular (aHR = 1.11[1.07, 1.15], *p* < 0.001), and ischemic heart disease (aHR = 1.10[1.08, 1.13], *p* < 0.001). Race and ethnicity were mostly not associated with increased risks (*p* > 0.05). aHRs with contemporary controls as a reference were similar to those with pre-pandemic cohort as a reference. We concluded that new incident cardiovascular disorders in COVID-19 patients, especially those hospitalized for COVID-19, were higher than those in controls. Identifying risk factors for developing new-onset cardiovascular disorders may draw clinical attention for the need for careful follow-up in at-risk individuals.

**Supplementary Information:**

The online version contains supplementary material available at 10.1038/s41598-024-82983-7.

## Introduction

The COVID-19 pandemic caused by the novel coronavirus, SARS-CoV-2, has rapidly evolved into one of the most significant global health crises of our time. Characterized by respiratory symptoms ranging from mild cough to severe acute respiratory distress syndrome, mounting evidence suggests that COVID-19 extends its impact far beyond the respiratory system. Emerging clinical reports have revealed an increased incidence of cardiovascular manifestations among COVID-19 patients, spanning from arrhythmias, inflammatory heart disease, thrombosis, cerebrovascular disorders, ischemic heart disease and other cardiac disorders (defined as cardiogenic shock, cardiac arrest, heart failure and non-ischemic cardiomyopathy) in individual without history of cardiovascular disorders^1,2^.

The mechanisms underlying COVID-19-associated cardiovascular complications are multifaceted and complex. Direct viral invasion of myocardial cells via angiotensin-converting enzyme 2 (ACE2) receptors, dysregulation of the renin-angiotensin-aldosterone system (RAAS), systemic inflammation, cytokine storm, endothelial dysfunction, and hypercoagulable states have been proposed as potential contributors to cardiac injury and dysfunction. Improved understanding of the incidence linking COVID-19 to cardiovascular disorders and the risk profiles are pivotal not only for elucidating the disease’s full clinical spectrum but also for guiding therapeutic strategies and public health interventions to mitigate cardiovascular complications in COVID-19 patients and improving overall clinical outcomes of patients with COVID-19.

The goal of this study was to evaluate the incidence of new-onset cardiovascular disorders up to 3.5 years post SARS-CoV-2 infection. The data came from the Montefiore Health System which serves a large low-income, diverse population in the Bronx and its environs, and was an epicenter of the early COVID-19 pandemic and subsequent surges of infections. Mathematical models were used to identify risk factors associated with developing these new-onset cardiovascular disorders.

## Materials and methods

### Data source

This study was approved by the Einstein-Montefiore Institutional Review Board (#2021–13658). Data originated from the Montefiore Health System consists of multiple hospitals and outpatient clinics located in the Bronx and its environs. Data were extracted as described previously^3–6^. All methods were carried out in accordance with relevant guidelines and regulations, including those of stated in the “Declaration of Helsinki.”

From March 11, 2020 to July 1, 2023, there were a total of 56,400 patients with a polymerase-chair-reaction COVID-19 positive test in the Montefiore Health System and 1,093,904 patients without a COVID-19 positive test (denoted as COVID-19 negative). The first date of positive COVID-19 results or the first visit to the Montefiore Health System after March 1st, 2020, was used as the index date for the COVID-19 and non-COVID-19 cohorts, respectively. Patients who died 30 days from the index date and who did not have at least one inpatient, outpatient or ER visit more than 30 days post index date were excluded. The follow-up time was up to 3.5 years post infection. COVID-19 positive cohort had a follow-up time of 430 ± 288 days (mean ± standard deviation) and non-COVID-19 cohort had a follow up time of 615 ± 375 days.

A historical cohort was also constructed following the same parameters with a study period from January 1, 2018 to March 1, 2020. For comparisons between the COVID-19 cohort and historical cohort, the contemporary cohort was limited to March 1, 2020 to May 1, 2022 such as the follow-up time was similar.

Demographic data (e.g., age, sex, ethnicity, race) and medical conditions including hypertension, hyperlipidemia, asthma, cancer, chronic obstructive pulmonary disease (COPD), chronic kidney disease (CKD), type 2 diabetes mellitus, and smoking history were tabulated. Cardiovascular conditions included arrhythmias, inflammatory heart disease, thrombosis, cerebrovascular disorders, ischemic heart disease and other cardiac disorders (defined as cardiogenic shock, cardiac arrest, heart failure and non-ischemic cardiomyopathy). OMOP concept ids and groupings are shown in Supplementary Table 1.

### Outcomes

The primary outcome was incidence of new-onset major adverse cardiovascular event (MACE) which was defined as all-cause mortality or incidence of any of the cardiovascular conditions (defined above) between 30 days after and up to 3.5 years following the index visit. For each cardiovascular condition, we examined the first chronological entry of a corresponding ICD-10 code for that condition relative to thirty days after a patient’s index date (to account for possible delayed entry into the medical record) to determine if they were in the ‘pre-existing’ population or if they were in the at-risk population during the follow-up period. Patients who already had a specific pre-existing cardiovascular condition as outcome were excluded from the Cox regression models for that specific condition.

### Statistical analysis

Statistical analyses were performed using Python’s Sklearn and Statsmodels libraries and SAS. Group differences in percentages for categorical variables were tested using the Pearson χ^2^ test, and the Student’s t-test were used for continuous variables.

Cumulative incidence functions for new-onset cardiovascular outcomes were calculated for Covid-19 status and stratified by ethnicity and race groups. Survival analysis for time to new-onset cardiovascular outcomes were performed using a Cox proportional hazard model. COVID-19 status, age, sex, ethnicity, CKD, COPD, asthma and cancer were included in the model as covariates. Hypertension, diabetes, smoking status, obesity and hyperlipidemia were adjusted for using inverse probability weighting in the Cox model (as opposed to including these as confounders) as they showed marked differences between groups. The other comorbidities which showed smaller group differences were treated as confounders in the model. Hazard ratios (HR) and their 95% confidence intervals (CI) were estimated and tested for significance. P-value < 0.05 was considered statistically significant.

## Results

There were 41,446 COVID-19 positive and 621,020 contemporary COVID-19 negative patients returned to our health system at least once 30 days post index date. Table 1 summarizes the demographics and comorbidities grouped by COVID-19 status. COVID-19 positive cohort was older, more Hispanic and more Blacks compared to non-COVID-19 cohort (*p* < 0.001). COVID-19 positive cohort had fewer male (*p* < 0.001). COVID-19 positive cohort had a higher prevalence of pre-existing hypertension, CKD, hyperlipidemia, COPD, asthma, cancer, diabetes, smoking, and obesity compared to non-COVID-19 cohort (*p* < 0.001). COVID-19 cohort has a greater prevalence of pre-existing history of arrhythmias, inflammatory heart disease, thrombosis, cerebrovascular disorders, other cardiac disorders and ischemic heart disease.

**Table 1:** Characteristics of patients with COVID-19 and without. Frequencies and percentages for categorical variables between cohorts of were compared using Chi-squared tests. Continuous variables were compared using Student’s t-test. Abbreviations: CKD, chronic kidney disease. COPD, Chronic obstructive pulmonary disease. MACE, major adverse cardiovascular event.

	COVID+ *N* = 41,446	COVID- *N* = 621,020	*P* Value
Demographics N (%)			
Age, yrs, mean (± SD)	45.5 (24.3)	42 (24.6)	< 0.001
Male	16,549 (39.9%)	263,808 (42.5%)	< 0.001
Hispanic	17,795 (42.9%)	219,628 (35.4%)	< 0.001
White	3505 (8.5%)	64,814 (10.4%)	< 0.001
Black	12,388 (29.9%)	156,503 (25.2%)	< 0.001
Other	7758 (18.7%)	180,075(29.0%)	< 0.001
Pre-existing Comorbidities N (%)			
Hypertension	16,859 (40.7%)	132,348 (21.3%)	< 0.001
CKD	5780 (13.9%)	28,088 (4.5%)	< 0.001
Hyperlipidemia	10,993 (26.5%)	64,938 (10.5%)	< 0.001
COPD	2944 (7.1%)	13,216 (2.1%)	< 0.001
Asthma	9191 (22.2%)	67,969 (10.9%)	< 0.001
Cancer	3245 (7.8%)	22,215 (3.6%)	< 0.001
Diabetes	10,003 (24.1%)	64,274 (10.3%)	< 0.001
Smoking	6958 (16.8%)	71,422 (11.5%)	< 0.001
Obesity	14,877 (36.0%)	139,982 (22.5%)	< 0.001
Pre-existing CVD N (%)			
Arrhythmias	6854 (16.6%)	36,389 (5.9%)	< 0.001
Inflammatory Heart Disease	80 (0.2%)	342 (0.01%)	< 0.001
Thrombosis	2122 (5.1%)	8420 (1.4%)	< 0.001
Cerebrovascular	1925 (4.6%)	8991 (1.5%)	< 0.001
Other Cardiac Disorder	3612 (8.7%)	15,638 (2.5%)	< 0.001
Ischemic Heart Disease	5493 (13.3%)	31,420 (5.1%)	< 0.001

Table 2 shows cardiovascular outcomes grouped by COVID-19 status. COVID-19 positive patients had higher new incidence of arrhythmias (6.4% vs. 4.6%, *p* < 0.001), inflammatory heart disease (0.2% vs. 0.1%, *p* < 0.001), thrombosis (2.7% vs. 1.2%, *p* < 0.001), cerebrovascular disorders (2.0% vs. 1.4%, *p* < 0.001), other cardiac disorders (3.5% vs. 2.4%, *p* < 0.001), and ischemic heart disease (5.1% vs. 3.8%, *p* < 0.001). COVID-19 positive patients had a higher incidence of any cardiovascular outcomes (14.1% vs. 9.9%, *p* < 0.001) and MACE (14.7% vs. 10.2%, *p* < 0.001) than non-COVID-19 patients.

**Table 2:** Outcomes of patients with COVID-19 and without. Frequencies and percentages for categorical variables between cohorts were compared using Chi-squared tests.

	COVID+	COVID-	*P* Value
Outcomes			
Arrhythmias	6.4% (2225/34592)	4.6% (27183/584631)	< 0.001
Inflammatory Heart Disease	0.2% (70/41366)	0.1% (391/620678)	< 0.001
Thrombosis	2.7% (1053/39324)	1.2% (7238/612600)	< 0.001
Cerebrovascular	2.0% (798/39521)	1.4% (8369/612029)	< 0.001
Other Cardiac Disorder	3.5% (1330/37834)	2.4% (14182/605382)	< 0.001
Ischemic Heart Disease	5.1% (1822/35953)	3.8% (22354/589600)	< 0.001
Composite Outcomes			
MACE	6076 (14.7%)	63,303 (10.2%)	< 0.001
Any cardiovascular outcome	5825 (14.1%)	61,682 (9.9%)	< 0.001

**Fig. 1:**
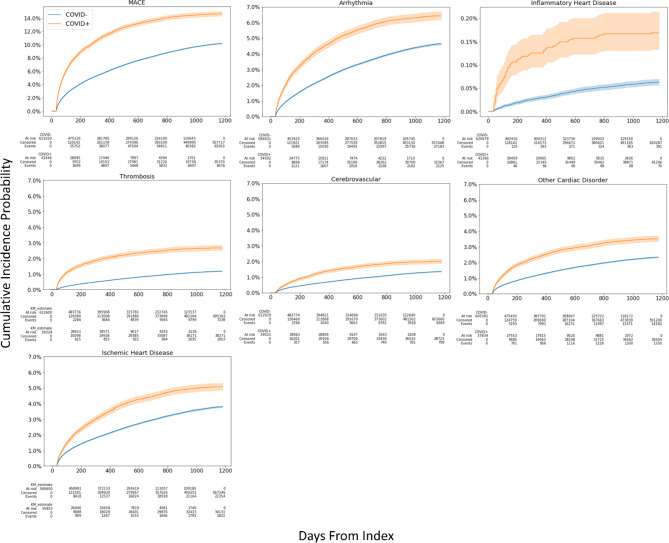
Cumulative incidence curves for cardiovascular outcomes with COVID-19 compared to non-COVID-19 patients. These are raw incidences that have not been adjusted for covariates. Shaded regions are 95% confidence intervals.

Figure 1 illustrates the cumulative incidence functions for developing cardiovascular outcomes among COVID-19 positive and negative patients. COVID-19 positive patients had a higher MACE incidence compared to non-COVID-19 patients (*p* < 0.05). Patients with COVID-19 had a higher incidence of developing arrhythmias, inflammatory heart disease, cerebrovascular disease, other cardiac disorders, thrombosis, and ischemic heart than non-COVID-19 patients (*p* < 0.05).

Table 3 shows the cumulative incidence of the cardiovascular outcomes stratified by ethnicity and race up to 3.5 years post index date. There were no significant differences for all cumulative incidences due to ethnicity nor race (*p* > 0.05), except in arrhythmias and other heart diseases between Hispanic and non-Hispanic patients, and in other heart diseases between White and Black patients (*p* < 0.05). Note that cumulative incidence of all cardiovascular outcomes was higher in COVID + compared to COVID- cohort for each of the racial and ethnic subgroups (*p* < 0.001).

**Table 3:** Cumulative incidences for cardiovascular outcomes with COVID-19 patients stratified by ethnicity and race. These are raw incidences that have not been adjusted for covariates. * *p* < 0.05 and ** *p* < 0.01 between ethnic groups and between racial groups. Note that all incidences between COVID + and COVID- were also significantly different in each subgroup (*p* < 0.001).

	MACE	Arrhythmias	Inflammatory heart disease	Cerebrovascular	Other Cardiac Disorder	Thrombosis	Ischemic heart disease
Hispanic	14.7%	6.9%**	0.14%	1.9%	3.3%*	2.7%	4.9%
Non-Hispanic	14.6%	6.1%**	0.19%	2.1%	3.7%*	2.7%	5.2%
White	16.5%	7.0%	0.24%	2.5%	4.3%*	3.5%	5.6%
Black	17.3%	7.5%	0.26%	2.5%	5.1%*	2.8%	6.4%

Table 4 summarizes the adjusted hazard ratios for COVID-19 status for developing new-onset cardiovascular diseases, along with covariates that included age, sex, ethnicity, CKD, COPD, asthma, and cancer. Hospitalized COVID-19 positive patients had significantly higher risk of developing MACE (aHR 2.29, 95% CI [2.27, 2.31], *p* < 0.001), arrhythmias (aHR 2.54 [2.50, 2.58], *p* < 0.001), inflammatory heart disease (aHR 5.34 [4.79, 5.96], *p* < 0.001), cerebrovascular (aHR 2.05 [2.00, 2.11], *p* < 0.001), other cardiac disorders (aHR 2.31 [2.26, 2.35], *p* < 0.001), thrombosis (aHR 4.25 [4.15, 4.36], *p* < 0.001), and ischemic heart disease (aHR 1.89 [1.86, 1.92], *p* < 0.001). Non-hospitalized COVID-19 positive patients also had slightly higher risk of developing MACE (aHR 1.04, 95% CI [1.03, 1.06], *p* < 0.001), arrhythmias (aHR 1.10 [1.08, 1.12], *p* < 0.001), inflammatory heart disease (aHR 2.29 [2.03, 2.59], *p* < 0.001), cerebrovascular (aHR 1.11 [1.07, 1.15], *p* < 0.001), and ischemic heart disease (aHR 1.10 [1.08, 1.13], *p* < 0.001). Non-hospitalized COVID-19 positive patients had a slightly lower risk of developing other cardiac disorders (aHR 0.89 [0.87, 0.92], *p* < 0.001) or thrombosis (aHR 0.92 [0.89, 0.96], *p* < 0.001). Overall, the HRs for hospitalized COVID-19 were higher than those for the non-hospitalized COVID-19. The HRs for the other covariates are also shown.

**Table 4:** Multivariable adjusted hazard ratios of hospitalized and non-hospitalized COVID-19 status and comorbidities for developing new-onset cardiovascular diseases. Values in parentheses are 95% confidence levels. * *p* < 0.05, ** *p* < 0.01, *** *p* < 0.001.

	MACE	Arrhythmias	Inflammatory Heart Disease	Cerebrovascular	Other Cardiac Disorder	Thrombosis	Ischemic Heart Disease
COVID Hospitalized vs. COVID-	2.29 (2.27,2.31)***	2.54 (2.50,2.58)***	5.34 (4.79,5.96)***	2.05 (2.00,2.11)***	2.31 (2.26,2.35)***	4.25 (4.15,4.36)***	1.89 (1.86,1.92)***
COVID Non-hospitalized vs. COVID-	1.04 (1.03,1.06)***	1.10 (1.08,1.12)***	2.29 (2.03,2.59)***	1.11 (1.07,1.15)***	0.89 (0.87,0.92)***	0.92 (0.89,0.96)***	1.10 (1.08,1.13)***
Age	1.03 (1.03,1.03)***	1.02 (1.02,1.02)***	0.99 (0.99,0.99)***	1.04 (1.04,1.04)***	1.05 (1.05,1.05)***	1.03 (1.03,1.03)***	1.05 (1.05,1.05)***
Gender	1.37 (1.36,1.38)***	1.28 (1.27,1.30)***	1.01 (0.93,1.08)	1.31 (1.28,1.35)***	1.60 (1.58,1.63)***	1.41 (1.38,1.44)***	1.64 (1.62,1.66)***
Hispanic	1.03 (1.02,1.04)***	1.11 (1.09,1.12)***	0.69 (0.64,0.75)***	0.89 (0.87,0.92)***	0.92 (0.91,0.94)***	1.00 (0.98,1.02)	0.98 (0.97,1.00)*
CKD	1.49 (1.47,1.50)***	1.60 (1.57,1.62)***	2.16 (1.92,2.42)***	1.40 (1.36,1.45)***	2.24 (2.19,2.28)***	1.34 (1.30,1.37)***	1.59 (1.56,1.62)***
COPD	1.48 (1.47,1.51)***	1.61 (1.58,1.65)***	0.90 (0.75,1.08)	1.29 (1.24,1.34)***	1.84 (1.80,1.89)***	1.42 (1.38,1.47)***	1.60 (1.56,1.63)***
Asthma	1.00 (0.99,1.01)	1.06 (1.04,1.08)***	1.14 (1.04,1.25)**	1.03 (0.99,1.06)	0.95 (0.93,0.97)***	0.94 (0.92,0.97)***	1.02 (1.00,1.04)*
Cancer	1.22 (1.21,1.24)***	1.22 (1.19,1.24)***	1.69 (1.49,1.91)*	1.05 (1.02,1.09)**	0.98 (0.95,1.00)	1.64 (1.60,1.68)***	0.87 (0.85,0.90)***

It is possible that pandemic circumstances, in addition to SARS-CoV-2 per se, could affect outcomes. We thus compared the results with historical (pre-pandemic) data as the reference. Supplementary Table 2 summarizes the demographics and comorbidities for the non-COVID-19 group compared to the historical cohort. The profiles of the contemporary non-COVID-19 cohort and the historical cohort were similar, although many variables showed significant group differences because of large sample sizes. Supplementary Table 3 shows the adjusted HRs of Hospitalized COVID-19 positive versus historical control with MACE (adjusted HR 1.91, 95% CI [1.86, 1.96], *p* < 0.001), arrhythmias (aHR 2.17 [2.08, 2.27], *p* < 0.001), inflammatory heart disease (aHR 4.63 [3.31, 6.46], *p* < 0.001), cerebrovascular (aHR 1.67 [1.54, 1.81], *p* < 0.001), other cardiac disorders (aHR 1.99 [1.88, 2.11], *p* < 0.001), thrombosis (aHR 3.52 [3.29, 3.78, *p* < 0.001), and ischemic heart disease (aHR 2.00 [1.90, 2.11], *p* < 0.001). These aHRs were similar to those with the contemporary non-COVID-19 controls as reference. Adjusted HRs for non-hospitalized COVID positive versus historical control with MACE (adjusted HR 0.81, 95% CI [0.78, 0.84], *p* < 0.001), arrhythmias (aHR 0.87 [0.83, 0.93], *p* < 0.001), inflammatory heart disease (aHR 1.86 [1.28, 2.71], *p* = 0.001), cerebrovascular (aHR 0.92 [0.82, 1.02], *p* = 0.11), other cardiac disorders (aHR 0.70 [0.64, 0.76], *p* < 0.001), thrombosis (aHR 0.68 [0.62, 0.76, *p* < 0.001), and ischemic heart disease (aHR 1.22 [1.14, 1.30], *p* < 0.001) were mixed, likely due to the low sample size of non-hospitalized COVID patients developing arrhythmias, cerebrovascular and other cardiac disorders (1.5-3%).

## Discussion

This study investigated the incidence of new-onset cardiovascular disorders of COVID-19 and non-COVID-19 patients up to 3.5 years post SARS-CoV-2 infection in a large diverse population in the Bronx which was an epicenter of the early COVID-19 pandemic and subsequent surges of infections. The major findings are COVID-19 positive patients, especially those hospitalized for COVID-19, had significantly higher risk of developing MACE, arrhythmias, inflammatory heart disease, cerebrovascular, other cardiac disorders, thrombosis, and ischemic heart disease compared to contemporary non-COVID-19 controls. There were no differences in risks for cardiovascular outcomes due to race status or ethnicity status. The adjusted hazard ratios of new-onset cardiovascular disorders with contemporary controls as reference were similar to those with historical cohort as reference.

Our COVID-19 adjusted hazard ratios for developing new-incident individual cardiovascular disorders ranged from 1.89 to 5.34 for the hospitalized cohort and 0.89 to 2.29 for the non-hospitalized cohort. The adjusted hazard ratio for inflammatory diseases as outcomes was particularly high (aHR = 5.34 in the hospitalized cohort and 2.29 in the non-hospitalized cohort), consistent with the pro-inflammatory responses associated with severe COVID-19^7,8^. The adjusted hazard ratio for thrombosis event an as outcome was also particularly high in the hospitalized cohort (aHR = 4.08), but not in the hospitalized cohort, consistent with the hypercoagulability associated with severe COVID-19^7,8^. Overall, the aHRs for hospitalized COVID-19 were markedly higher than those for the non-hospitalized COVID-19, suggesting these outcomes are associated with COVID-19 disease severity.

Our results on risks for cardiovascular disorders are in general agreement with the literature although most prior studies reported a comparatively shorter follow-up time and were not stratified by COVID-19 hospitalization status. Supplementary Table 4 summarizes the literature described below. Lim et al. evaluated at 1,790,097 patients from a Singapore claims database and found had increased risk (HR = 1.157 [1.069,1.252]) of new-incident cardiovascular, cerebrovascular, and other thrombotic complications after COVID-19 from September 2020 to November 2021 with ~ 1 year follow up^9^. Ortega-Paz et al. studied 4,427 patients 1 year post COVID-19 from Spain and Italy and found that COVID-19 patients had higher rates of arterial thrombotic events (aHR = 2.26 [1.02,4.99] *p* = 0.044), venous thromboembolism (aHR = 9.33 [2.93, 29.70] *p* = 0.001) and arrhythmias (aHR: 3.37, [1.35,8.46] *p* = 0.010), but no difference in cardiovascular death when compared to a COVID-19 negative cohort^10^. Raisi-Estabragh et al. evaluated at 17,871 people from the UK Biobank cases for up to 1 year follow up and found that hospitalized COVID-19 patients had extremely high risk of venous thromboembolism (HR = 27.6 [14.5, 52.3] *p* < 0.0001), heart failure (HR = 21.6 [10.9, 42.9] *p* < 0.0001) and stroke (HR = 17.5 [5.26, 57.9] *p* < 0.0001) compared to propensity matched controls^11^. Lo Re et al. evaluated 90-day risk of thromboembolism in 85,637 patients hospitalized with COVID-19 (April 2020 to May 2021) versus 8,269 influenza patients (October 2018 to April 2019) from the US Food and Drug Administration Sentinel System. They found the risk of venous thromboembolism was significantly higher among patients with COVID-19 before vaccine availability (aHR = 1.60 [1.43, 1.79]) and during vaccine availability (aHR = 1.89 [1.68, 2.12]) in comparison to influenza patients^12^. Rezel-Potts et al. evaluated 428,650 COVID-19 patients from 2020 to 2021 (1 year follow up and patients from first wave) without diabetes or cardiovascular disease from the Aurum database and found that adjusted rate ratios of COVID-19 risk for cardiovascular outcomes was 5.82 [4.82, 7.03] (*p* < 0.001) at 4 weeks post-infection and 0.80 [0.73, 0.88] (*p* < 0.001) at 13 to 52 weeks post-infection compared with propensity-matched controls over the same period^13^. Roi-Teeuw et al. evaluated at 862,189 patients from the UK’s Clinical Practice Research Datalink and found that increased incidence rates up to 60 days after SARS-CoV-2 infection for venous and arterial cardiovascular events and new-onset atrial fibrillation, but not for inflammatory heart disease or heart failure, with the highest rate for venous events (13 per 1000 person-years)^14^. Terenschencko et al. evaluated at 65,585 patients from the Oregon Health and Science University Health system for up to 6 months post COVID-19 and found that COVID-19 had an adjusted hazard ratio of 1.71 [1.06, 2.78] *p* = 0.029) for cardiovascular death or morbidity, such as acute heart failure, acute coronary syndrome, non-ST-elevation myocardial infarction, incident stroke or transient ischemic attack, another acute or new CV outcome prompting health-care utilization^15^. Wan et al. investigated post-acute outcomes of 7,139 patients from the UK Biobank for 18 months post COVID-19 and found that COVID-19 patients had significant hazard ratios for any new onset of heart failure, stroke or coronary heart disease (aHR = 5.0 [3.0, 8.1]) compared to a historical cohort of 71,314 patients^16^. Wang et al. studied 690,892 participants from the TriNetX database for incidental cardiovascular outcomes for 1 year post infection with propensity score-matched controls. COVID-19 survivors were associated with increased risks of cerebrovascular diseases were higher in the COVID-19 survivors than in the controls (HR ranged from 1.5 to 2.8)^17^. Wiemken et al. evaluated at 1,357,518 COVID-19 patients in the US Health Verity Real-Time Insights and Evidence database from April 2020 to May 2021. They found that there was an increased risk for cardiovascular events in ICU (aHR = 1.80 [1.71, 1.89]) or non-ICU hospitalization (aHR = 1.28 [1.24, 1.33]) vs. non-hospitalized patients^18^. Xie et al. evaluated 153,760 patients with COVID-19 from the Saint Louis VA system for new onset cardiovascular disorders > 30 days post COVID-19 with 1 year follow up and found that there was an increased risk of incident cerebrovascular disorders, dysrhythmias, ischemic and non-ischemic heart disease, pericarditis, myocarditis, heart failure and thromboembolic disease^2^. Battistoni et al. studied 31,764 subjects with a diagnosis of COVID-19 from 2020 to 2022 (up to 2-year follow-up) in Italy and found that new adverse cardiovascular and cerebrovascular events odd ratios was 1.73 [1.53, 1.94] (*P* < 0.001) compared to propensity score-matched controls from 2017 to 2019. Incidence was the highest in the first-year post COVID-19 and remained elevated throughout the 3 years^19^.

The novelty of our study is that it included SARS-CoV-2 infections from March 11, 2020, to July 1, 2023 and our follow-up time was up to 3.5 years post infection in a large low-income, diverse population in the Bronx which was an epicenter of the early COVID-19 pandemic and subsequent surges of infections. We also stratified by COVID-19 hospitalization status. More COVID-19 patients during early pandemic likely experienced more severe COVID-19 in general before effective treatments and COVID-19 vaccines became available^20,21^. Thus, it is likely our odds ratios are likely at the lower ranges. However, underserved populations could be more susceptible to new incident cardiovascular disorders as this population could have higher prevalence of pre-existing medical conditions. Different COVID-19 testing rates across the pandemic and across different geographic regions could also affect incidence of outcomes^4,11,22^. Different experimental designs and patient profiles, among others, could contribute to diverse findings. Another novelty is that we found that that COVID-19 patients developed cardiovascular disorders at a faster rate within 1-year post infection and slowed down over time as indicated by cumulative incidence curves, supporting the notion that outcome is associated with COVID-19 status. Overall, the HRs for hospitalized COVID-19 were higher than those for the non-hospitalized COVID-19.

### Race and ethnicity differences

There were significant differences in cumulative incidences of arrythmias and other heart diseases between Hispanic and non-Hispanic patients with COVID-19, and in other heart diseases between White and Black patients with COVID-19 (*p* < 0.05). These were not adjusted for covariates. Janus et al. reported different results in the CDC WONDER database, namely, Black patients experienced higher incidence in myocardial infarction, stroke and heart failure of myocardial infarction (MI), stroke, and heart failure from 2020 to 2021 than White patients^23^. Song et al. found that Hispanic and Black patients had more excess cardiovascular death compared to White patients in the same database from 2020 to 2022^24^. Wadhera et al. also reported Black, Asian, and Hispanic populations experienced a larger relative increase in deaths than the non-Hispanic White population (interaction term, *P* < 0.001) in the NCHS dataset from March to August 2020^25^. These studies, however, looked at population level data and not on the individual level. Other factors, such as socioeconomic status, could also contribute to worse outcomes^26,27^.

When we stratified by individual cardiovascular disorders, there were scattered differences due to race and/or ethnicity. However, incident individual cardiovascular disorders were low (0.5-2%) and our population consisted of larger proportions of Blacks and Hispanics, and thus, our study was thus underpowered to address potential racial and ethnicity differences in individual cardiovascular disorders.

### Mechanisms of increased susceptibility to cardiovascular disorders

SARS-CoV-2 could cause cardiac dysfunction as there is evidence that SARS-CoV-2 directly infects cardiac cells via the ACE2 receptors^28^. Persistent activation of RAAS and endothelial injury have been reported among patients with COVID-19 and both are associated with blood pressure elevation. In addition, consequences of severe COVID-19 including systemic hypoxia, acute respiratory distress, hypercoagulation, sepsis, inflammation, metabolic stress, and cytokine storm may stress the cardiovascular system that could lead to adverse cardiovascular outcomes^7^. There is evidence of direct effects of SARS-CoV-2 on the myocardium, thrombotic damage to vessels or endothelium, persistent inflammation^7,8^. Chronic autoimmune response to cardiac antigens could also contribute to new cardiovascular disorders^29^.

### Pandemic circumstance

Other factors contributing to new incident cardiovascular disorders following COVID-19 may include the effects of isolation, psychosocial stress, reduced physical activity, unhealthy diet, weight gain and difficulty in assessing care during the early pandemic^30^. We thus compared results with respect to the historical cohort. Our historical cohort had a slightly higher incidence of arrhythmias, thrombosis, and other cardiac disorders than our contemporary cohort without COVID-19, but lower than our contemporary cohort with COVID-19. Although there were statistical differences between groups due to large sample sizes, most of these differences were unlikely to be clinically significant. A possible explanation is that there are improvements in population health over time. The overall HRs however were similar whether the contemporary or historical cohorts was used as the reference, further supporting the notion that COVID-19 status (especially those who were hospitalized for COVID-19) is significant and important risk factor for developing new incident cardiovascular disorders.

### Limitations

Our study has several limitations. This is a single health system study with multiple hospitals and outpatient clinics, but larger studies are needed to improve generalizability. Our population is diverse, and our findings might not be generalizable to populations that are less diverse. Only patients who returned to our health system were included. Those who returned to our health system (although for any medical reasons, including regular checkup) might have a higher burden of comorbidities compared to those did not return. However, such loss to follow-up affects both COVID-19 positive and negative cohorts and should not alter our overall conclusions. COVID-19 vaccination status was not reliable as individuals might have had vaccines elsewhere, and thus outcomes were not analyzed with respect to COVID-19 vaccination status. As with any retrospective studies, there could be additional unintentional patient selection bias and residual confounders that could not be accounted for^31–33^.

## Conclusion

The incidence of new-onset cardiovascular disorders in patients with COVID-19 is higher than in patients without COVID-19. Identifying risk factors for developing new-onset cardiovascular disorders may draw clinical attention for the need for careful follow-up in at-risk individuals post–COVID-19 infection.

## Electronic Supplementary Material

Below is the link to the electronic supplementary material.

Supplementary Material 1

Supplementary Material 2

Supplementary Material 3

Supplementary Material 4

## Data Availability

Data is available at https://doi.org/10.5281/zenodo.12631418.
