# Supplementary material for: New-onset cardiovascular diseases post SARS-CoV-2 infection in an urban population in the Bronx
Source: Sci Rep. 2024 Dec 28;14:31451. doi: 10.1038/s41598-024-82983-7 (PMC11682409; doi:10.1038/s41598-024-82983-7)
Supplement: Supplementary file 1 — Supplementary Material 1 [file 41598_2024_82983_MOESM1_ESM.docx]

**Supplementary Table 1** Cardiovascular diseases and OMOP concept ids

| Cerebrovascular Disorders | |
| --- | --- |
| 443454 | Cerebral infarction |
| 4108356 | Cerebral infarction due to embolism of cerebral arteries |
| 4110189 | Cerebral infarct due to thrombosis of precerebral arteries |
| 4110190 | Cerebral infarction due to embolism of precerebral arteries |
| 4110192 | Cerebral infarction due to thrombosis of cerebral arteries |
| 4111714 | Cerebral infarction due to cerebral venous thrombosis, non-pyogenic |
| 45767658 | Cerebral infarction due to thrombosis of middle cerebral artery |
| 45772786 | Cerebral infarction due to embolism of middle cerebral artery |
| 46270031 | Cerebral infarction due to occlusion of precerebral artery |
| 46273649 | Cerebral infarction due to occlusion of basilar artery |
| 432923 | Subarachnoid hemorrhage |
| 4049659 | Subcortical hemorrhage |
| 4108952 | Subarachnoid hemorrhage from carotid siphon and bifurcation |
| 4110186 | Intracerebral hemorrhage, multiple localized |
| 4111708 | Subarachnoid hemorrhage from vertebral artery |
| 4111709 | Non-traumatic subdural hemorrhage |
| 4144154 | Non-traumatic intracerebral ventricular hemorrhage |
| 4148906 | Spontaneous subarachnoid hemorrhage |
| 4176892 | Cortical hemorrhage |
| 4319328 | Brain stem hemorrhage |
| 42535425 | Spontaneous hemorrhage of cerebral hemisphere |
| 43530674 | Spontaneous cerebellar hemorrhage |
| 43530727 | Spontaneous cerebral hemorrhage |
| Arrhythmias | |
| 313217 | Atrial fibrillation |
| 313792 | Paroxysmal tachycardia |
| 314665 | Atrial flutter |
| 433225 | Ventricular flutter |
| 437894 | Ventricular fibrillation |
| 444070 | Tachycardia |
| 4089462 | Ventricular premature complex |
| 4103295 | Ventricular tachycardia |
| 4111552 | Re-entry ventricular arrhythmia |
| 4141360 | Chronic atrial fibrillation |
| 4154290 | Paroxysmal atrial fibrillation |
| 4169095 | Bradycardia |
| 4232691 | Permanent atrial fibrillation |
| 4232697 | Persistent atrial fibrillation |
| 4275423 | Supraventricular tachycardia |
| 36712986 | Atypical atrial flutter |
| 36714994 | Typical atrial flutter |
| 44784217 | Cardiac arrhythmia |
| 45768480 | Longstanding persistent atrial fibrillation |
| Inflammatory Heart Disease | |
| 312653 | Acute myocarditis |
| 314383 | Myocarditis |
| 4231274 | Viral myocarditis |
| 4331309 | Myocarditis due to infectious agent |
| 320116 | Acute pericarditis |
| 4138837 | Pericarditis |
| 4217075 | Infectious pericarditis |
| 4289908 | Viral pericarditis |
| 4181182 | Chronic adhesive pericarditis |
| 4203625 | Chronic constrictive pericarditis |
| Ischemic Heart Disease | |
| 319844 | Acute ischemic heart disease |
| 315286 | Chronic ischemic heart disease |
| 315296 | Preinfarction syndrome |
| 316427 | Aneurysm of coronary vessels |
| 321318 | Angina pectoris |
| 443563 | Arteriosclerosis of coronary artery bypass graft |
| 764123 | Atherosclerosis of coronary artery without angina pectoris |
| 4108215 | Coronary thrombosis not resulting in myocardial infarction |
| 4124683 | Silent myocardial ischemia |
| 4127089 | Coronary artery spasm |
| 36712779 | Chronic total occlusion of coronary artery |
| 36712982 | Unstable angina co-occurrent and due to coronary arteriosclerosis |
| 36712983 | Angina co-occurrent and due to coronary arteriosclerosis |
| 37115756 | Dissection of coronary artery |
| 37312532 | Coronary arteriosclerosis in artery of transplanted heart |
| 40481132 | Arteriosclerosis of coronary artery bypass graft of transplanted heart |
| 40481919 | Coronary atherosclerosis |
| 40482638 | Arteriosclerosis of autologous vein coronary artery bypass graft |
| 40482655 | Arteriosclerosis of nonautologous coronary artery bypass graft |
| 43021857 | Arteriosclerosis of autologous arterial coronary artery bypass graft |
| 4198141 | Post infarct angina |
| 438172 | Atrial septal defect due to and following acute myocardial infarction |
| 4108219 | Rupture of chordae tendinae due to and following acute myocardial infarction |
| 4108220 | Rupture of papillary muscle as current complication following acute myocardial infarction |
| 4108678 | Hemopericardium due to and following acute myocardial infarction |
| 4108679 | Rupture of cardiac wall without hemopericardium as current complication following acute myocardial infarction |
| 4108680 | Thrombosis of atrium, auricular appendage, and ventricle due to and following acute myocardial infarction |
| 4270024 | Acute non-ST segment elevation myocardial infarction |
| 4296653 | Acute ST segment elevation myocardial infarction |
| 4329847 | Myocardial infarction |
| 37309626 | Myocardial infarction due to demand ischemia |
| 43020460 | Acute ST segment elevation myocardial infarction involving left anterior descending coronary artery |
| 46270162 | Acute ST segment elevation myocardial infarction due to left coronary artery occlusion |
| 46270163 | Acute ST segment elevation myocardial infarction due to right coronary artery occlusion |
| 4110961 | Generalized ischemic myocardial dysfunction |
| Other Cardiac Disorder | |
| 319835 | Congestive heart failure |
| 439696 | Hypertensive heart and renal disease with (congestive) heart failure |
| 439846 | Left heart failure |
| 443580 | Systolic heart failure |
| 443587 | Diastolic heart failure |
| 444101 | Hypertensive heart failure |
| 4004279 | High output heart failure |
| 4014159 | Chronic right-sided heart failure |
| 4195785 | Right heart failure secondary to left heart failure |
| 4229440 | Chronic congestive heart failure |
| 4242669 | Biventricular congestive heart failure |
| 37309625 | Acute on chronic right-sided congestive heart failure |
| 40479192 | Chronic systolic heart failure |
| 40479576 | Chronic diastolic heart failure |
| 40480602 | Acute on chronic systolic heart failure |
| 40481043 | Acute on chronic diastolic heart failure |
| 44782719 | Chronic combined systolic and diastolic heart failure |
| 44782733 | Acute on chronic combined systolic and diastolic heart failure |
| 4233424 | Acute right-sided heart failure |
| 40480603 | Acute systolic heart failure |
| 40481042 | Acute diastolic heart failure |
| 44782718 | Acute combined systolic and diastolic heart failure |
| 316139 | Heart Failure |
| 321042 | Cardiac arrest |
| 4172822 | Cardiac arrest due to cardiac disorder |
| 4309332 | Cardiac arrest as a complication of care |
| 198571 | Cardiogenic shock |
| Thrombosis | |
| 440417 | Pulmonary embolism |
| 40479606 | Septic pulmonary embolism |
| 44782732 | Chronic pulmonary embolism |
| 193512 | Embolism and thrombosis of the renal vein |
| 199837 | Portal vein thrombosis |
| 313761 | Central retinal vein occlusion |
| 435887 | Antepartum deep vein thrombosis |
| 438820 | Postpartum deep phlebothrombosis |
| 762443 | Thrombosis of superficial vein of penis |
| 4149782 | Thrombosis of vein of lower limb |
| 4179911 | Axillary vein thrombosis |
| 4207615 | Thrombosis of vein of trunk |
| 4258295 | Embolism from thrombosis of vein of distal lower extremity |
| 40481089 | Embolism from thrombosis of vein of lower extremity |
| 43531681 | Acute deep vein thrombosis of lower limb |
| 44782743 | Acute deep venous thrombosis of popliteal vein |
| 44782744 | Acute deep venous thrombosis of tibial vein |
| 44782747 | Acute deep venous thrombosis of femoral vein |
| 44782751 | Acute deep venous thrombosis of axillary vein |
| 44782752 | Acute deep venous thrombosis of internal jugular vein |
| 44782754 | Chronic thrombosis of superficial vein of upper extremity |
| 44782755 | Chronic deep venous thrombosis of internal jugular vein |
| 44782759 | Chronic thrombosis of subclavian vein |
| 44782760 | Acute thrombosis of superficial vein of upper extremity |
| 44782762 | Acute thrombosis of subclavian vein |
| 46273489 | Thrombosis of vein of upper limb |
| 77310 | Deep vein phlebitis and thrombophlebitis of the leg |
| 313219 | Phlebitis and thrombophlebitis |
| 439838 | Thrombophlebitis migrans |
| 4143293 | Superficial thrombophlebitis in puerperium |
| 4146460 | Superficial thrombophlebitis in pregnancy |
| 45757143 | Postpartum septic thrombophlebitis |
