# Supplementary material for: New-onset cardiovascular diseases post SARS-CoV-2 infection in an urban population in the Bronx
Source: Sci Rep. 2024 Dec 28;14:31451. doi: 10.1038/s41598-024-82983-7 (PMC11682409; doi:10.1038/s41598-024-82983-7)
Supplement: Supplementary file 2 — Supplementary Material 2 [file 41598_2024_82983_MOESM2_ESM.docx]

**Supplementary Table 2.** Characteristics of patients with COVID-19, without COVID-19 and a historical cohort over a 2-year follow-up time. Frequencies and percentages for categorical variables between cohorts of were compared using Chi-squared tests. Continuous variables were compared using Student’s t-test. Abbreviations: CKD, chronic kidney disease. COPD, Chronic obstructive pulmonary disease. MACE, major adverse cardiovascular event. * p<0.05, ** p<0.01, *** p<0.001.

|  | COVID+ | COVID- | Historical Cohort | P value  COVID+ vs COVID- | P value  COVID+ vs Historical | P value  COVID- vs Historical |
| --- | --- | --- | --- | --- | --- | --- |
| Demographics N (%) | 29526 | 558032 | 64541 |  |  |  |
| Age, yrs, mean (±SD) | 45.8 (23.4) | 42.4 (24.4) | 44.4 (24.8) | *** | *** | *** |
| Male | 12003 (40.7%) | 235010 (42.1%) | 26821 (41.6%) | *** | ** | ** |
| Hispanic | 12740 (43.1%) | 201706 (36.1%) | 23838 (36.9%) | *** | *** | *** |
| White | 2547 (8.6%) | 57764 (10.4%) | 7073 (11.0%) | *** | *** |  |
| Black | 8893 (30.1%) | 146373 (26.2%) | 16866 (26.1%) | *** | *** |  |
| Other | 5346 (18.1%) | 152189 (27.3%) | 16764 (26.0%) | *** | *** | * |
| **Pre-existing Comorbidities N (%)** |  |  |  |  |  |  |
| Hypertension | 11623 (39.4%) | 127691 (22.9%) | 15580 (24.1%) | *** | *** | *** |
| CKD | 3932 (13.3%) | 27564 (4.9%) | 3977 (6.2%) | *** | *** | *** |
| Hyperlipidemia | 7398 (25.1%) | 63505 (11.4%) | 6676 (10.3%) | *** | *** | *** |
| COPD | 1922 (6.5%) | 12932 (2.3%) | 1905 (3.0%) | *** | *** | *** |
| Asthma | 6315 (21.4%) | 66300 (11.9%) | 7607 (11.8%) | *** | *** |  |
| Cancer | 2089 (7.1%) | 21429 (3.8%) | 3150 (4.9%) | *** | *** | *** |
| Diabetes | 7024 (23.8%) | 62250 (11.2%) | 8142 (12.6%) | *** | *** | *** |
| Smoking | 4675 (15.8%) | 68121 (12.2%) | 8502 (13.2%) | *** | *** | *** |
| Obesity | 10647 (36.1%) | 132928 (23.8%) | 13739 (21.3%) | *** | *** | *** |
| **Outcomes** |  |  |  |  |  |  |
| Arrhythmias | 4.5% (1137/24994) | 3.3% (17072/522262) | 3.3% (2017/60601) | *** | *** |  |
| Inflammatory Heart Disease | 0.12% (38/29468) | 0.04% (245/557694) | 0.04% (24/64500) | *** | *** |  |
| Thrombosis | 2.2% (622/28119) | 0.8% (4508/549713) | 1.0% (617/63340) | *** | *** | *** |
| Cerebrovascular | 1.3% (373/28278) | 0.9% (5084/549157) | 0.9% (569/63542) | *** | *** |  |
| Other Cardiac Disorder | 2.6% (696/27145) | 1.7% (8977/542579) | 1.7% (1073/62239) | *** | *** |  |
| Ischemic Heart Disease | 3.5% (921/25985) | 2.6% (13474/527157) | 2.2% (1309/60853) | *** | *** | *** |
| **Composite Outcomes** |  |  |  |  |  |  |
| Any cardiovascular outcome | 3069 (10.4%) | 39669 (7.1%) | 4560 (7.1%) | *** | *** |  |
| MACE | 3364 (11.4%) | 41865 (7.5%) | 5331 (8.3%) | *** | *** | *** |
