# Supplementary material for: New-onset cardiovascular diseases post SARS-CoV-2 infection in an urban population in the Bronx
Source: Sci Rep. 2024 Dec 28;14:31451. doi: 10.1038/s41598-024-82983-7 (PMC11682409; doi:10.1038/s41598-024-82983-7)
Supplement: Supplementary file 3 — Supplementary Material 3 [file 41598_2024_82983_MOESM3_ESM.docx]

**Supplementary Table 3.** Multivariable adjusted hazard Ratios of COVID-19 and comorbidities for developing new-onset cardiovascular diseases. The potential confounding effects of hypertension, hyperlipidemia, diabetes, smoking and obesity were adjusted using inverse probability weighting. * p<0.05, ** p<0.01, *** p<0.001.

|  | **MACE** | **Arrhythmias** | **Inflammatory Heart Disease** | **Cerebrovascular** | **Other Cardiac Disorder** | **Thrombosis** | **Ischemic Heart Disease** |
| --- | --- | --- | --- | --- | --- | --- | --- |
| **COVID Hospitalized vs Historical** | 1.91 (1.86,1.96)*** | 2.17 (2.08,2.27)*** | 4.63 (3.31,6.46)*** | 1.67 (1.54,1.81)*** | 1.99 (1.88,2.11)*** | 3.52 (3.29,3.78)*** | 2.00 (1.90,2.11)*** |
| **COVID Non-hospitalized vs Historical** | 0.81 (0.78,0.84)*** | 0.87 (0.83,0.93)*** | 1.86 (1.28,2.71)** | 0.92 (0.82,1.02) | 0.70 (0.64,0.76)*** | 0.68 (0.62,0.76)*** | 1.22 (1.14,1.30)*** |
| **Age** | 1.03 (1.03,1.03)*** | 1.02 (1.02,1.02)*** | 0.99 (0.98,0.99)*** | 1.05 (1.04,1.05)*** | 1.04 (1.04,1.04)*** | 1.03 (1.03,1.03)*** | 1.05 (1.05,1.05)*** |
| **Male** | 1.33 (1.30,1.36)*** | 1.20 (1.15,1.24)*** | 1.55 (1.23,1.96)*** | 1.33 (1.23,1.42)*** | 1.53 (1.45,1.60)*** | 1.36 (1.28,1.43)*** | 1.53 (1.46,1.60)*** |
| **Hispanic** | 1.01 (0.99,1.03) | 1.08 (1.04,1.12)*** | 0.61 (0.47,0.77)*** | 1.07 (0.99,1.15) | 0.87 (0.83,0.92)*** | 1.00 (0.95,1.05) | 1.03 (0.99,1.08) |
| **CKD** | 1.81 (1.76,1.86)*** | 1.89 (1.80,1.98)*** | 2.35 (1.68,3.29)*** | 1.74 (1.61,1.89)*** | 2.48 (2.34,2.62)*** | 1.60 (1.50,1.71)*** | 1.99 (1.89,2.10)*** |
| **COPD** | 1.40 (1.36,1.45)*** | 1.40 (1.32,1.49)*** | 1.03 (0.62,1.71) | 1.47 (1.33,1.63)*** | 1.59 (1.48,1.71)*** | 1.21 (1.11,1.32)*** | 1.64 (1.53,1.75)*** |
| **Asthma** | 1.13 (1.10,1.16)*** | 1.19 (1.14,1.25)*** | 1.19 (0.90,1.57) | 0.95 (0.87,1.05) | 1.39 (1.31,1.48)*** | 1.08 (1.00,1.15)* | 1.06 (0.99,1.12) |
| **Cancer** | 1.46 (1.42,1.51)*** | 1.50 (1.43,1.59)*** | 0.65 (0.38,1.11) | 1.15 (1.04,1.27)** | 1.21 (1.13,1.29)*** | 1.75 (1.63,1.88)*** | 1.06 (1.00,1.13) |
