# Supplementary material for: New-onset cardiovascular diseases post SARS-CoV-2 infection in an urban population in the Bronx
Source: Sci Rep. 2024 Dec 28;14:31451. doi: 10.1038/s41598-024-82983-7 (PMC11682409; doi:10.1038/s41598-024-82983-7)
Supplement: Supplementary file 4 — Supplementary Material 4 [file 41598_2024_82983_MOESM4_ESM.docx]

**Supplementary Table 4**. Summary of papers presented in discussion section.

| **Study** | **Source of data** | **Sample Size** | **Date of study** | **Follow Up Post Index Date** | **Findings** |
| --- | --- | --- | --- | --- | --- |
| Lim | Singapore Ministry of Health database | 1,790,097 | Sep 2020 to Nov 2021 | 300 days median | Increased risk (HR=1.157 [1.069,1.252]) of new-incident cardiovascular, cerebrovascular, and other thrombotic complications after COVID-19 |
| Ortega-Paz | CV COVID-19 registry, 17 centers from Spain and Italy | 4,427 | Feb 2020 to Jul 2021 | 1 year | Higher rates of arterial thrombotic events (aHR=2.26 [1.02,4.99] p=0.044), venous thromboembolism (aHR=9.33 [2.93, 29.70] p=0.001) and arrhythmias (aHR: 3.37, [1.35,8.46] p=0.010), but no difference in cardiovascular death when compared to a COVID-19 negative cohort |
| Raisi-Estabragh | UK Biobank | 17,871 | Mar 2020 to Mar 2021 | 1 year | COVID-19 patients had high risk of venous thromboembolism (HR=27.6 [14.5, 52.3] p<0.0001), heart failure (HR=21.6 [10.9, 42.9] p<0.0001) and stroke (HR=17.5 [5.26, 57.9] p<0.0001) compared to propensity matched controls |
| Lo Re | US Food and Drug Administration Sentinel System database | 85,637 | Apr 2020 to May 2021 | 90 day | Risk of venous thromboembolism was significantly higher among patients with COVID-19 before vaccine availability (aHR=1.60 [1.43, 1.79]) and during vaccine availability (aHR=1.89 [1.68, 2.12]) in comparison to influenza patients |
| Rezel-Potts | Clinical Research Practice Link Aurum database | 428,650 | Jan 2020 to Jan 2022 | 1 year | Adjusted rate ratios of COVID-19 risk for cardiovascular outcomes was 5.82 [4.82, 7.03] (p<0.001) at 4 weeks post-infection and 0.80 [0.73, 0.88] (p<0.001) at 13 to 52 weeks post-infection compared with propensity-matched controls over the same period |
| Roi-Teeuw | Clinical Research Practice Link Aurum database, patients | 862,189 | Jan 2020 to Sep 2021 | 180 days | Increased incidence rates up to 60 days after SARS-CoV-2 infection for venous and arterial cardiovascular events and new-onset atrial fibrillation, but not for inflammatory heart disease or heart failure, with the highest rate for venous events (13 per 1000 person-years |
| Terenschencko | Oregon Health and Science University Health | 65,585 | Mar 2020 to Sep 2020 | 6 months | COVID-19 had an adjusted hazard ratio of 1.71 [1.06, 2.78] p=0.029) for cardiovascular death or morbidity, such as acute heart failure, acute coronary syndrome, non-ST-elevation myocardial infarction, incident stroke or transient ischemic attack, another acute or new CV outcome prompting health-care utilization |
| Wan | UK Biobank | 7,139 | Mar 2020 to Nov 2020 | 18 months | COVID-19 patients had significant hazard ratios for any new onset of heart failure, stroke or coronary heart disease (aHR=5.0 [3.0, 8.1]) compared to a historical cohort |
| Wang | TriNetX database | 690,892 | Mar 2020 to Jan 2022 | 1 year | COVID-19 survivors were associated with increased risks of cerebrovascular diseases were higher in the COVID-19 survivors than in the controls (HR ranged from 1.5 to 2.8) |
| Wiemken | US Health Verity Real-Time Insights & Evidence database | 1,357,518 | Apr 2020 to May 2021 | 30 days | COVID-19 patients had increased risk for cardiovascular events in ICU (aHR=1.80 [1.71, 1.89]) or non-ICU hospitalization (aHR=1.28 [1.24, 1.33]) vs non-hospitalized patients |
| Xie | Saint Louis VA system | 153,760 | Mar 2020 to Mar 2021 | 1 year | COVID-19 patients had increased risk of incident cerebrovascular disorders, dysrhythmias, ischemic and non-ischemic heart disease, pericarditis, myocarditis, heart failure and thromboembolic |
| Battistoni | COMEGEN database, Naples, Italy | 31,764 | Jan 2020 to Jan 2022 | 2 year | New adverse cardiovascular and cerebrovascular events odd ratios was 1.73 [1.53, 1.94] (P<0.001) compared to propensity score-matched controls from 2017 to 2019. Incidence was the highest in the first-year post COVID-19, and remained elevated afterwards |
